# Effect of different biochars on acid soil and growth parameters of rice plants under aluminium toxicity

**DOI:** 10.1038/s41598-020-69262-x

**Published:** 2020-07-23

**Authors:** Rajpal Shetty, Nagabovanalli Basavarajappa Prakash

**Affiliations:** 1 Department of Soil Science and Agricultural Chemistry, University of Agricultural Sciences, GKVK, Bengaluru, India; 2 Department of Plant Physiology, Faculty of Natural Sciences, Comenius University, Bratislava, Slovakia

**Keywords:** Pollution remediation, Environmental impact

## Abstract

Biochar is known to decrease the soil acidity and in turn enhance the plant growth by increasing soil fertility. Major objective of the present work was to understand the effect of biochar treatment on alleviation of soil aluminium (Al) toxicity and its role in enhancing plant growth parameters. Soil incubation study was conducted to understand the effect of biochar (*Eucalyptus* wood, bamboo, and rice husk) on soil pH, soluble and exchangeable Al in soil with and without Al addition. Another independent pot experiment with rice crop (*Oryza sativa* L. *var*. *Anagha*) was carried out for 120 days to examine the effect of biochars on soil properties and growth parameters of rice plants. Wood biochar application to soil at 20 t ha^−1^ was found to be highly consistent in decreasing soil acidity and reducing soluble and exchangeable Al under both studies. We conclude that wood biochar at higher dose performed better in reducing soluble and exchangeable Al in comparison to other biochars indicating its higher ameliorating capacity. However, rice husk biochar was effective under Al untreated soil, indicating the role of Si-rich biochars in enhancing plant growth.

## Introduction

Soil acidity in many parts of the world poses a significant challenge to crop productivity. Around 30% of the total land area in the world and more than 50% of the world’s potentially arable area fall under acidic soils^1–3^. Approximately 30% of arable land in India are acidic in nature, resulting in low crop yield^3^. Acid soils have low pH values (< 5.5 or 6) and are usually associated with the severe aluminium (Al) toxicity to plants. Aluminium is mainly in the form of insoluble silicate or oxide in neutral soils (pH = 6.5—7.5). However, low soil pH (< 5) leads to the solubilization of Al, primarily to the phytotoxic form of Al^3+^ in soil solution^4,5^.

Aluminium toxicity has been reported to cause direct inhibition of root elongation and further interfere with uptake of plant nutrients^6,7^. Aluminium toxicity can therefore be considered a primary limiting factor in acid soils for plant growth and development. Consequently, focusing on worldwide acid soil remediation is crucial to enhance crop yield and thus alleviating world hunger. Liming has been the prominent approach for amending acid soil. However, biochar application as soil amendment has been receiving lot of attention, for many reasons such as neutralizing acidity in soil, creating a carbon (C) sink to mitigate global warming, increasing soil water holding capacity, reducing greenhouse gas emissions and stabilizing mobile heavy metals, pesticides and other organic pollutants in soil^8-12^.

Biochar is carbon rich material obtained by pyrolysis of biomass with little or no oxygen^13^. Pyrolysis of plant biomass normally results highly alkaline biochar^13–15^. However, alkalinity varies with respect to feedstock properties used for the biochar production. Greater the alkalinity of biochar, greater is the reduction in acidity^16^. Addition of biochar to nutrient poor soil has been reported to enhance nutrient availability and increase plant biomass^17^. Application of peanut shell biochar to highly acidic red soil was reported to enhance growth in cabbage by reducing Al toxicity owing to increased soil pH and nutrient availability^18^. High surface charge density, large surface area and internal porosity, and presence of both polar and non-polar surface sites on biochar play a vital role in metal adsorption along with the liming effect^14^. Therefore, incorporation of biochar into acid soil could help to mitigate soil Al toxicity by decreasing soil exchangeable acidity, increasing soil exchangeable base cations, and thereby improving soil fertility. A lot of studies have been carried out on the impact of biochar on soil acidity^19–21^. However, very few studies have specifically focussed on the impact of biochar on soil Al content^18,22^.

The purpose of this study was to evaluate the impact of different biochar sources and rates on the extent of Al toxicity mitigation in acid soil under incubated condition. Additionally, an independent pot experiment was performed to investigate the performance of different biochar sources and rates on soil properties and on certain growth parameters of rice plants under acid soils.

## Results

### Incubation study: effect of biochar on soil pH, soluble and exchangeable Al

#### Soil without Al treatment

**Figure 1:**
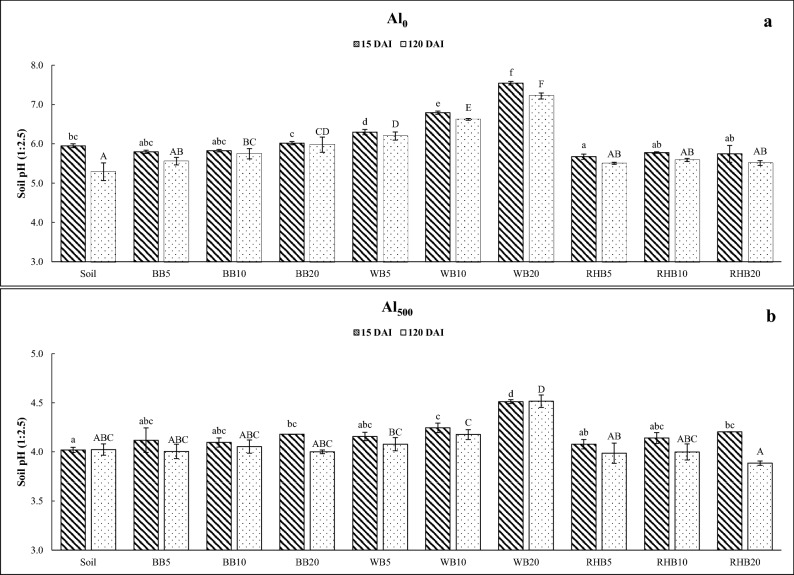
Incubation study: effect of different biochar treatments on soil pH. (**a**) Changes in soil pH in Al untreated soil (Al_0_: 0 mg kg^−1^) and (**b**) changes in soil pH in Al treated soil (Al_500_: 500 mg kg^−1^). BB: bamboo biochar. WB: wood biochar. RHB: rice husk biochar. DAI: days after incubation. Biochar rates in t ha^−1^. Each value represents the mean of three replicates ± standard error. Mean values marked with same letters do not differ significantly according to the Tukey’s HSD test at *p* ≤ 0.05.

Addition of wood biochar (WB) at all application rates (5, 10 and 20 t ha^−1^) led to a significant increase in soil pH at 15 days after incubation (DAI) (Fig. 1a). The addition of WB at all application rates, and the application of bamboo biochar (BB) at the rates of 10 and 20 t ha^−1^ led to an increase in soil pH after 120 days. However, application of rice husk biochar (RHB) did not increase the soil pH. Soluble and exchangeable Al were not detected under Al untreated soil.

#### Soil with 500 mg kg^−1^ Al treatment

Addition of WB at 10 and 20 t ha^−1^, BB and RHB at 20 t ha^−1^ significantly increased the soil pH compared to control at 15 DAI (Fig. 1b). Nonetheless, after 120 DAI only WB at 20 t ha^−1^ was found to significantly increase the soil pH compared to control.

**Figure 2:**
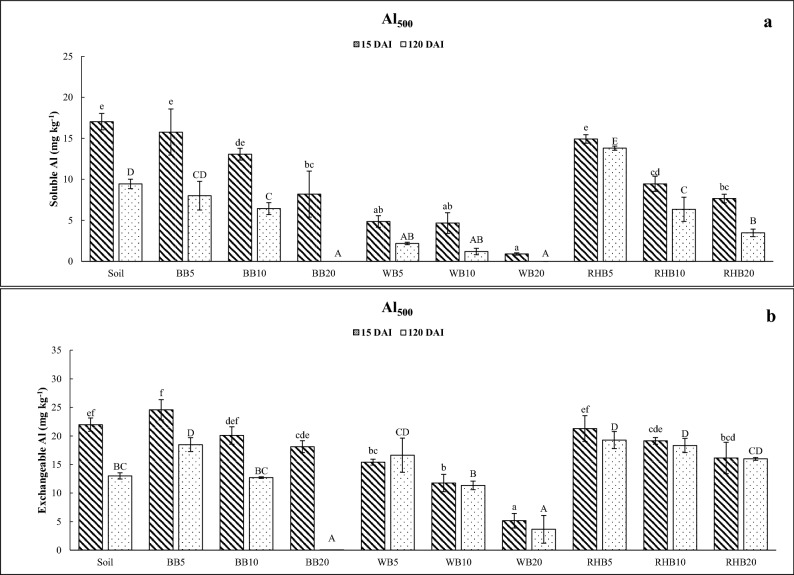
Incubation study: effect of different biochar treatments on the soluble and exchangeable Al content of soil. (**a**) Changes in soluble Al in Al treated soil (Al_500_: 500 mg kg^−1^) and (**b**) changes in exchangeable Al under Al treated soil (Al_500_: 500 mg kg^−1^). BB: bamboo biochar. WB: wood biochar. RHB: rice husk biochar. DAI: days after incubation. Biochar rates in t ha^−1^. Each value represents the mean of three replicates ± standard error. Mean values marked with same letters do not differ significantly according to the Tukey’s HSD test at *p* ≤ 0.05.

The addition of WB at all application rates, BB at the rate of 20 t ha^−1^, and RHB at the rates of 10 and 20 t ha^−1^ significantly decreased the soluble Al in soil at 15 DAI (Fig. 2a). Similar effect was noticed after 120 DAI except for BB and RHB at the rate of 5 t ha^−1^.

The addition of WB at all application rates, and RHB at 20 t ha^−1^ significantly decreased the exchangeable Al at 15 DAI compared to soil without biochar treatment (Fig. 2b). However, only WB and BB at the rate of 20 t ha^−1^ significantly decreased the exchangeable Al at 120 DAI.

### Pot study with rice crop: effect of biochar on soil properties after harvest

#### Soil without Al treatment

**Figure 3:**
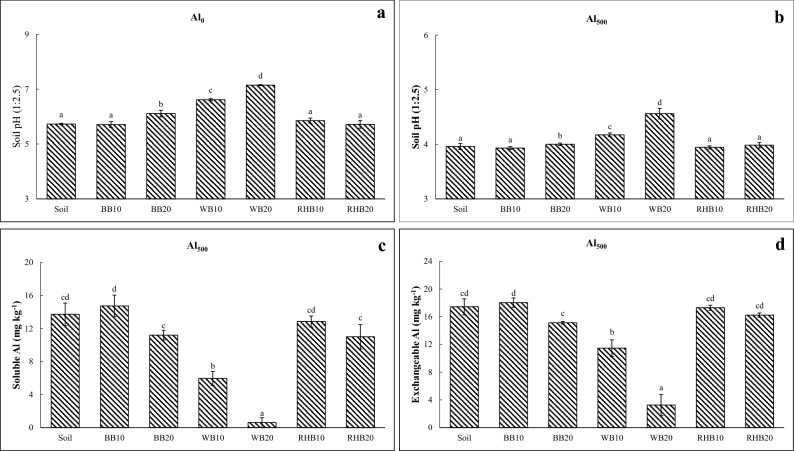
Pot study with rice crop: effect of different biochar treatments on the soil pH, soluble and exchangeable Al content of soil. (**a**) Changes in soil pH in Al untreated soil (Al_0_: 0 mg kg^−1^), (**b**) changes in soil pH in Al treated soil (Al_500_: 500 mg kg^−1^), (**c**) changes in soluble Al in Al treated soil (Al_500_: 500 mg kg^−1^) and (**d**) changes in exchangeable Al in Al treated soil (Al_500_: 500 mg kg^−1^). BB: bamboo biochar. WB: wood biochar. RHB: rice husk biochar. Biochar rates in t ha^−1^. Each value represents the mean of three replicates ± standard error. Mean values marked with same letters do not differ significantly according to the Tukey’s HSD test at *p* ≤ 0.05.

Addition of WB at all application rates (10 and 20 t ha^−1^), and the application of BB at the rate of 20 t ha^−1^ led to significant increase in the soil pH (Fig. 3a). Soil electrical conductivity (EC) was increased with the application of WB at 20 t ha^−1^ compared to other treatments (Table 1). Biochar treatments did not impact soil exchangeable acidity or exchangeable calcium (Ca). Interestingly, addition of biochars at all application rates increased the available silicon (Si) in soil, wherein significant increase to an extent of 119.21 mg kg^−1^ Si was found due to the addition of RHB at the rate of 20 t ha^−1^. However, there was no detectable quantities of soluble or exchangeable Al in soil, either due to the biochar addition or in control soil.

**Table 1:** Pot study with rice crop: effect of different biochar treatments on the selected soil properties under Al untreated (Al_0_: 0 mg kg^−1^) and Al treated soil (Al_500_: 500 mg kg^−1^).

Treatments	EC (dS m^−1^)		Exchangeable acidity (cmol (p +) kg^−1^)		Exchangeable Ca (cmol (p +) kg^−1^)		Available Si (mg kg^−1^)	
	Al_0_	Al_500_	Al_0_	Al_500_	Al_0_	Al_500_	Al_0_	Al_500_
Soil	0.45 ± 0.01 (ab)	0.95 ± 0.22 (a)	0.20 ± 0.05 (a)	3.16 ± 0.31 (d)	2.28 ± 0.09 (a)	1.90 ± 0.27 (a)	63.00 ± 4.95 (a)	44.92 ± 4.16 (ab)
BB 10 t ha^−1^	0.32 ± 0.14 (a)	1.05 ± 0.10 (ab)	0.18 ± 0.06 (a)	2.80 ± 0.13 (cd)	2.68 ± 0.60 (a)	2.54 ± 0.46 (ab)	70.42 ± 8.94 (ab)	47.00 ± 9.99 (ab)
BB 20 t ha^−1^	0.33 ± 0.05 (a)	1.52 ± 0.16 (bc)	0.27 ± 0.16 (a)	2.90 ± 0.28 (cd)	1.93 ± 0.24 (a)	3.03 ± 0.06 (bc)	88.17 ± 18.51 (bc)	36.71 ± 0.15 (a)
WB 10 t ha^−1^	0.42 ± 0.04 (ab)	1.57 ± 0.09 (c)	0.10 ± 0.00 (a)	1.88 ± 0.20 (b)	1.69 ± 0.59 (a)	4.14 ± 0.18 (d)	83.46 ± 4.98 (abc)	39.46 ± 3.29 (ab)
WB 20 t ha^−1^	0.52 ± 0.01 (b)	1.60 ± 0.15 (c)	0.12 ± 0.03 (a)	0.96 ± 0.08 (a)	3.02 ± 1.75 (a)	5.16 ± 0.21 (e)	95.17 ± 4.90 (cd)	46.38 ± 4.59 (ab)
RHB 10 t ha^−1^	0.34 ± 0.06 (a)	1.55 ± 0.27 (c)	0.15 ± 0.00 (a)	2.58 ± 0.08 (c)	2.29 ± 0.06 (a)	3.60 ± 0.56 (cd)	93.04 ± 1.38 (bc)	46.75 ± 4.25 (ab)
RHB 20 t ha^−1^	0.38 ± 0.03 (ab)	1.55 ± 0.15 (c)	0.15 ± 0.00 (a)	2.93 ± 0.06 (cd)	2.30 ± 0.08 (a)	3.56 ± 0.08 (cd)	119.21 ± 4.85 (d)	51.96 ± 2.47 (b)

BB: bamboo biochar, WB: wood biochar, RHB: rice husk biochar. Each value represents the mean of three replicates ± standard error. Mean values marked with same letters do not differ significantly according to the Tukey’s HSD test at *p* ≤ 0.05.

#### Soil with 500 mg kg^−1^ Al treatment

Soil pH in Al treated soil significantly increased by addition of WB at all application rates, and the application of BB at the rate of 20 t ha^−1^ (Fig. 3b). Increasing trend of soil EC was noticed by all the rates of biochar application. The highest increase in soil EC was observed with addition of WB at the rate of 20 t ha^−1^ compared to soil without biochar treatment (Table 1). Addition of biochar at all the rates decreased the soil exchangeable acidity, although highest reduction was noticed by the addition of WB at the rate of 20 t ha^−1^. Similarly, addition of WB at the rate of 20 t ha^−1^ significantly increased the exchangeable Ca in soil compared to other treatments. However, soil Si content was higher with addition of RHB at the rate of 20 t ha^−1^. Addition of WB at all application rates significantly reduced the soluble and exchangeable Al compared to all the treatments (Fig. 3c, d).

### Pot study with rice crop: effect of biochar on the growth parameters of rice crop

#### Soil without Al treatment

Application of RHB at the rate of 10 and 20 t ha^−1^ significantly increased the plant height, shoot and root dry weight compared to all the treatments (Table 2). However, biochar addition did not produce any significant effect on root length of rice crop grown in Al untreated soil.

**Table 2:** Pot study with rice crop: effect of different biochar treatments on the measured plant growth parameters under Al untreated (Al_0_: 0 mg kg^−1^) and Al treated soil (Al_500_: 500 mg kg^−1^).

Treatments	Plant height (cm)		Shoot dry weight (g)		Root dry weight (g)		Root length (cm)	
	Al_0_	Al_500_	Al_0_	Al_500_	Al_0_	Al_500_	Al_0_	Al_500_
Soil	87.50 ± 2.29 (ab)	71.03 ± 1.30 (a)	5.55 ± 0.36 (a)	2.88 ± 0.51 (a)	0.93 ± 0.06 (ab)	0.63 ± 0.09 (a)	27.66 ± 2.52 (a)	13.67 ± 0.58 (a)
BB 10 t ha^−1^	85.23 ± 2.47 (a)	74.50 ± 2.78 (ab)	5.38 ± 0.13 (a)	3.30 ± 0.74 (ab)	0.91 ± 0.04 (ab)	0.71 ± 0.06 (a)	33.00 ± 3.61 (a)	15.00 ± 3.00 (ab)
BB 20 t ha^−1^	87.87 ± 2.23 (abc)	78.15 ± 6.53 (ab)	5.53 ± 0.86 (a)	4.51 ± 1.12 (ab)	1.09 ± 0.17 (abc)	0.77 ± 0.24 (a)	34.33 ± 9.29 (a)	16.00 ± 6.00 (ab)
WB 10 t ha^−1^	87.86 ± 2.73 (abc)	80.58 ± 8.34 (ab)	5.44 ± 0.02 (a)	4.88 ± 0.97 (ab)	0.88 ± 0.12 (a)	0.88 ± 0.08 (a)	30.67 ± 5.13 (a)	19.67 ± 0.58 (ab)
WB 20 t ha^−1^	92.10 ± 3.20 (abc)	79.52 ± 5.41 (ab)	5.72 ± 0.22 (a)	5.40 ± 0.15 (b)	0.98 ± 0.10 (ab)	0.89 ± 0.21 (a)	32.33 ± 3.21 (a)	22.00 ± 2.65 (b)
RHB 10 t ha^−1^	96.47 ± 3.86 (c)	79.78 ± 6.33 (ab)	8.48 ± 0.97 (b)	3.59 ± 0.33 (ab)	1.26 ± 0.26 (bc)	0.65 ± 0.26 (a)	25.33 ± 0.58 (a)	18.00 ± 2.00 (ab)
RHB 20 t ha^−1^	95.55 ± 4.72 (bc)	88.32 ± 7.02 (b)	9.25 ± 0.83 (b)	5.42 ± 1.51 (b)	1.46 ± 0.08 (c)	0.68 ± 0.14 (a)	27.67 ± 2.52 (a)	19.33 ± 2.31 (ab)

BB: bamboo biochar, WB: wood biochar, RHB: rice husk biochar. Each value represents the mean of three replicates ± standard error. Mean values marked with same letters do not differ significantly according to the Tukey’s HSD test at *p* ≤ 0.05.

#### Soil with 500 mg kg^−1^ Al treatment

Aluminium addition to the soil decreased all the plant growth parameters measured (plant height, shoot and root dry weight, and root length) compared to plants grown in soil without Al addition. However, biochar treatments aided in improvement of those parameters. Plant height was significantly improved by the addition of RHB at the rate of 20 t ha^−1^. Addition of WB and RHB at the rate of 20 t ha^−1^ significantly increased the shoot dry weight. There was no significant change in root dry weight, even though biochar treatments increased root dry weight compared to soil without biochar treatment. However, biochar treatments increased the root length, wherein significant increase was noticed by the application of WB at the rate of 20 t ha^−1^.

## Discussion

### Soil pH

Application of WB increased the soil pH in both Al treated and untreated soils. Many studies have reported substantial increase in soil pH by the biochar treatment^15,17,20,21,23,24^. This has been attributed to the liming potential of biochar due to high inherent pH of biochar, base cation content, CaCO_3_ content and calcium carbonate equivalent (CCE)^20,21^. Increase in pH with the application of WB is due to high pH of WB and high EC indicating its higher soluble salts, greater CCE and Ca content compared to other biochars^12,13,17^. Biochar application might have resulted in neutralization of soil acidity by series of proton consumption reactions as reported by many studies^15,21^. However, consistent reduction in soil acidity by BB was only at 20 t ha^−1^, both in incubation and pot study with rice plants. Even, RHB was inconsistent in decreasing the soil pH, and in some cases, pH was lower than control. This can be due to lower production temperature, lower pH, and EC of RHB^25^. This shows that the pH of biochar is not the only parameter decreasing the soil acidity. Different biomass feedstocks and pyrolytic conditions mainly influences biochar properties. For example, biochar produced from leguminous feedstock can have higher liming potential than non-leguminous feedstocks^15,16^. In the present investigation, feedstocks from wood, bamboo and rice husk had varied properties. We assume that higher liming potential of wood biochar might be due to high uptake of basic cations from the *Eucalyptus* trees. Moreover, RHB had high content of N which might have aggravated the soil nitrification contributing to the acidity. McBeath and Smernik^26^ reported faster mineralization and degradability of biochar produced at lower temperature. Similarly, Harris et al.^27^ observed higher mineralization of C in case of peanut hull biochar than pine chip biochar as it contains three times higher ash and approximately 12 g kg^−1^ more aliphatic compounds than pine chip biochar. Similarly, Yuan et al.^16^ in an incubation study demonstrated that an increase in soil pH with application of different crop residue was inversely proportional to their nitrogen (N) content. Therefore, possible mineralization and subsequent nitrification of RHB might be the reason for reduction in the soil pH in our study. Interestingly, all the pH values in the present investigation had a decreasing trend at the end of incubation. This might be due to decomposition of soil organic matter and other microbial processes such as nitrification contributing to the soil acidity. Such decrease in pH with incubation time was also noticed by Hass et al.^28^ and Wan et al.^29^. However, from the pot study with rice plants having higher soil quantity compared to incubation, it was clearly noticed that WB application rates consistently decreased pH both under Al treated and untreated soils compared to other biochar treatments.

### Soluble and exchangeable Al

Recent studies indicate that liming effect of biochar, adsorption properties and the surface adsorption and co-precipitation of Al with silicate particles to fix Al in soil^22,30,31^. Similarly, biochar application to Al treated soil in our study reduced the soluble and exchangeable Al. These results are consistent with several reports suggesting that biochar application has potential to decrease Al toxicity in soil^22,30,32^. Increase in biochar rates can multiply its liming effect leading to strong adsorption of Al monomers and further conversion of toxic Al^3+^ to less toxic Al(OH)_3_ and Al(OH)_4_^-^ species^22^.

Addition of WB and increase in its rate of application to Al treated soil significantly decreased the soluble and exchangeable Al at the end of the incubation period (Fig. 2). Dang et al.^33^ reported that *Eucalyptus* biochar prepared at 550° C had high proton, Al, and iron (Fe) binding capacity due to various kinds of functional groups. Additionally, larger porosity of WB used in our study indicates higher surface area which in turn might have improved adsorption capacity^25^. Qian et al.^22^ reported that the biochar’s adsorption and precipitation capacity plays greater role in Al toxicity alleviation compared to its alkalinity. Complexation of Al with organic hydroxyl and carboxyl groups or the surface adsorption and co-precipitation of Al with silicate particles in biochar are reported as effective mechanisms.

There was no significant reduction in soluble and exchangeable Al of soil by treating BB or RHB with the application of less than 10 t ha^−1^ at the end of both studies. Sometimes, the soluble and exchangeable Al was noticed to be higher than control. We suppose that the substantial decrease in soil pH led to release of inherent Al of BB and RHB and thus increased the availability of Al in soil. Bamboo biochar did not have the same effect as WB even though it had properties like high pH and Si content. Similarly, RHB did not prove to be as effective as WB even with highest Si content and nearly equal CCE. This suggests that the alleviation of Al toxicity by biochar depends on its feed stock materials, pyrolysis temperature and other properties which play interdependent role.

### Soil EC

Significant increase in soil EC with biochar application has often been reported in previous studies^12,17,21,34,35^. In our study, biochar application to Al untreated soil did not increase the soil EC. However, increasing trend in soil EC was noticed by biochar application under Al treated soil. Highest increase was in case of WB at 20 t ha^−1^ compared to control. Natural increase in soil EC due to the increase in soluble salt content brought by increase in soil Al might be one of the reasons. Further, basic cations present in biochar might have solubilised into the soil solution under highly acidic pH resulting net increase of soil EC.

### Exchangeable acidity

The reduction of exchangeable acidity of soil clearly indicates the ability of biochars to decrease the exchangeable Al^3+^ and H^+^ in soil solution. The largest reduction was in case of WB at 10 and 20 t ha^−1^ and RHB at 10 t ha^−1^. Similar results were reported by Yamato et al.^36^, wherein wood biochar (bark of *Acaia mangium*) application was found to reduce the exchangeable acidity significantly under highly acidic soil.

### Exchangeable Ca

Free bases present in biochar such as Ca, magnesium (Mg) and potassium (K) can be readily released into to the soil solution resulting in net increase of soil pH and exchangeable bases. Such observations were also noticed by Lehmann et al.^19^ and Chan et al.^35^. In contrast, biochar treatments in our study did not affect exchangeable Ca under Al untreated soil. However, significant increase in Ca was noticed by biochar treatments under Al treated soil. Similar trend was reported by Silber et al.^37^ in a study on kinetics of release of Ca from corn straw biochar that the Ca release was increased as pH decreased. The largest increase in the present investigation was in case of WB at 20 t ha^−1^ in both Al treated and untreated soil indicating its liming potential.

### Available Si

Addition of biochar at all application rates increased the available Si in Al untreated soil. However, available Si content was decreased in Al treated soil. Increase in available Si might be due to increase in soil pH by alkaline biochar in turn enhancing Si availability and by the additional input of inherent Si from biochar^38^. Inversely, low pH by the addition of Al to soil might have hindered the dissolution and release of Si from biochar and further complexation of Si with free Al in soil solution. Highest increase in Si was observed by the addition of RHB at 20 t ha^−1^ under both Al treated and untreated soil, owing to its inherent Si content. Similar studies with rice husk and straw biochars have proven to significantly increase Si availability to plants^30,38-40^.

### Plant growth parameters

Aluminium decreased all the plant growth parameters due to its phytotoxicity, which mainly inhibits the root elongation. Previous studies have noticed the effect of Al toxicity on plants such as inhibiting root elongation, biomass reduction, oxidative stress, disrupting the function of the plasma membrane and cell wall, disordering calcium homeostasis, altering the signal transduction pathways and DNA damage^7^. However, biochar application has shown to aid in improvement of those parameters. Improvement of plant height and productive tillers per plant by biochar application has been reported previously^11,41^. Similarly, addition of WB and RHB in our study have shown profound influence in increasing plant height, shoot and root dry weight and further reducing the root length.

Biochar from rice husk might have had higher advantage due to its substantial amount of Si content. Beneficial effect of Si on the rice plant height and number of tillers are well known^42-44^. Recent studies have reported that Si rich biochars obtained by the feedstocks such as rice husk and straw have high potential in increasing soil available Si^30,32,38,39^. Such phytoavailable Si might further assist in decreasing Al toxicity to plants by forming the Al-Si compounds in the soil solution and in root tip^30^. On the other hand, WB at higher dose improved root length significantly compared to soil with Al treatment alone. This might be due to increase in the soil pH providing favourable plant growth conditions and reducing metal toxicity by complexation and precipitation of toxic Al^3+^. In addition, large boron (B) content in WB might have decreased Al^3+^ accumulation to cell wall and thus decreasing Al phytotoxicity. Many studies have reported that the optimum supply of B in soil can enhance root elongation, decrease Al content in root apoplast, prevents root injury and further promotes plant growth under Al toxicity^45-47^.

Numerous factors can act individually or simultaneously to increase plant growth under biochar treatments; viz., decrease in soluble Al and Fe, increase in soil pH, balanced and slow release of nutrients, increased plant available water and improved microbial activity. RHB being a rich source of majority of essential nutrients promoted the plant growth, in addition to its high CEC, Si and ash content. But especially under Al treated soil, WB performed better by decreasing the Al phytotoxicity to plant roots and thus improving the plant growth attributes. Hence, our study indicates the performance of WB as Al toxicity ameliorant, whereas RHB as a potential soil nutrient supplement with special reference to Si.

## Conclusions

Application of wood biochar to amend Al toxicity in the present study was proved to be very effective. It had a consistent effect on increasing soil pH and decreasing the soluble and exchangeable Al. Thus, improved the soil nutrient availability resulting in the higher yield of experimental plants. On the other hand, application of rice husk biochar enhanced the plant growth most significantly in Al untreated soil, even though it did not match the performance of wood biochar with respect to Al reduction in Al treated soil. Possible reason for higher yield with application of rice husk biochar may be due to higher availability of nutrients and especially benefits of Si on rice crop. However, low performance of RHB in Al treated soil due to its low pH, EC, Ca, and B content. It is evident that the ameliorating effect of biochar is dependent on its pH value, pyrolysis temperature and feedstock materials. Hence, categorization of potential biochars for amending the Al toxic soil must be recommended based on their individual properties. Long term field trials on Al contaminated soils must be conducted to further evaluate the effectiveness of biochar as an amendment for sustainable remediation of Al contaminated acidic soils.

## Materials and methods

### Soil

Soil samples were collected from a depth of 30 cm in the farm field (N 12º 57′ 02.4″, E 075º 58′ 18.0″) located in the Hassan district of Karnataka, South India representing southern dry zone and taxonomically classified as *Rhodic Paleustalfs*. Collected soil was air dried and sieved using 2 mm sieve. Particle size determination by international pipette method^48^ revealed that the soil is of sandy loam texture consisting of 68.95% sand, 8.65% silt and 22.40% clay. Some of the initial properties of the soil are shown in Table 3.

**Table 3:** Initial values of soil parameters under the study.

Parameters	Mean
pH (1:2.5)	5.96
EC (dSm^−1^)	0.15
Exchangeable acidity (m. eq H^+^ 100 g^−1^)	0.25
Exchangeable Ca (m. Equation 100 g^−1^)	3.02
Exchangeable Mg (m. Equation 100 g^−1^)	2.14
Available Si (mg kg^−1^)	70.04
Soluble Al (mg kg^−1^)	nd*
Exchangeable Al (mg kg^−1^)	nd*

*nd—not detected.

### Biochar

Biochars were produced from pyrolysis of *Eucalyptus* wood, WB (Pointec Pencil Energy Pvt. Ltd., Attibele, Bengaluru) and BB at around 550 °C (Indo-French Centre, IISc, Bengaluru), while RHB was produced at around 400 °C by the conventional mound method^49^ in the laboratory of plant mineral nutrition, Soil Science Department, GKVK, Bengaluru. The biochars were finely powdered and sieved using a 0.2 mm sieve. Biochar samples were combusted at about 950 °C in CNS analyser for estimation of total contents of N, C, and sulphur (S) (Dry Combustion, CNS, LECO). Acid digested biochar samples were used to quantify the total nutrient content using atomic absorption spectrophotometer (Perkin—Elmer AAnalyst 700 AAS)^50^. Some of the physicochemical properties and nutrient composition of biochars were measured (Table 4).

**Table 4:** Some of the physico-chemical properties and nutrient contents of biochars under the study.

	Wood biochar	Bamboo biochar	Rice husk biochar
**Physical properties**			
Bulk density (kg m^-3^)	0.31	0.61	0.53
Particle density (kg m^-3^)	1.32	1.43	1.76
Porosity (%)	73.83	57.27	69.85
Maximum water holding capacity (%)	213.31	93.71	131.41
Colour	5 YR 2/1	5 YR 2/1	5 YR 2/1
Ash content (%)	8.8	6.9	39.4
**Chemical properties**			
pH (1:5)	10.5	10.03	7.39
Electrical condcutivity (1:5) (dS m^−1^)	4.99	1.98	1.62
Cation exchange capacity [cmol (p^+^) kg^−1^]	26.25	23.43	38.63
Calcium carbonate equivalent (%)	31.00	27.50	30.50
**Nutrient contents**			
Carbon (per cent)	72.5	75.5	39.33
Nitrogen (per cent)	0.13	0.38	0.78
Phosphorus (per cent)	0.15	0.06	0.24
Potassium (per cent)	1.47	0.86	0.96
Calcium (per cent)	2.3	0.32	0.36
Magnesium (per cent)	0.48	0.38	0.31
Sulphur (per cent)	0.07	0.1	0.05
Sodium (per cent)	0.1	0.03	0.05
Silicon (per cent)	2.03	5.22	32.5
Zinc (mg kg^−1^)	23.9	58.6	63
Copper (mg kg^−1^)	36.6	32.7	31
Manganese (mg kg^−1^)	630.8	393.5	554
Iron (mg kg^−1^)	553.7	692.7	775.3
Boron (mg kg^−1^)	24.42	3.44	8.36

### Identification of phytotoxic levels of aluminium on aerobic rice

A preliminary experiment was conducted to identify the phytotoxic levels of Al concentration in the soil. The soil was treated with eight levels of Al concentration (0, 500, 1,000, 2000, 6,000, 8,000, 12,000 and 16,000 mg kg^−1^) using aluminium sulphate (Al_2_(SO_4_)_3_.16H_2_O) and incubated for a month maintained at field capacity moisture regime. After one-month, sub samples of 200 g soil of each was used in separate pots and replicated thrice for growing rice to identify the toxic levels of Al. Further, the comparison between direct seeded and transplanted seedling (10 days old) was done to compare the germination and seedling growth until 15 days. In direct seed method, the rice seedlings could germinate and grow up to two leaf stage under 500 mg kg^−1^ Al, while no seedlings could survive at more than 1,000 mg kg^−1^ of Al in transplanted method. Based on these results, 500 mg kg^−1^ of Al was selected for inducing Al toxicity in soil to understand the effect of biochar on decreasing Al toxicity under incubation study. Further, a pot study with rice plant was conducted with the same level of Al to know the effect of biochar on plant growth under Al phytotoxicity.

### Incubation study

Experimental setup was divided into two conditions based on Al addition to the soil viz., soil without and with Al (500 mg kg^−1^ of Al). Pots (11 and 7 cm of outer and inner diameter, respectively and 10.5 cm height) were filled with 200 g of treated soil with three replications. The soil samples were mixed with three types of biochar viz., BB, WB and RHB at 5, 10 and 20 t ha^−1^ respectively. The soil without biochar treatment served as control for each experiment. Moisture content was maintained at field capacity by weighing the pots periodically and weight loss due to evaporation was made up by adding distilled water. The treatments were duplicated twice for destructive soil sampling at 15 and at 120 days of incubation. Soil samples were analysed for the pH, soluble and exchangeable Al.

### Pot study with rice plants

The pot experiment with rice plants had two sets viz., soil without and with Al (500 mg kg^−1^ of Al). These soil samples were mixed with three types of biochar viz., BB, WB and RHB at 10 and 20 t ha^−1^ respectively. Five kg of treated soil was filled in each pot (22 and 14 cm of outer and inner diameter, respectively and 20 cm height). Three seeds of aerobic rice, variety *Anagha* were directly sown in the pot. The moisture level was maintained at field capacity to assure aerobic method of rice cultivation^51^. Nitrogen was applied in 3 splits viz., 50% as basal, 25% each at 30 and 60 days after sowing. While 100% of phosphorus (P) and K were applied along with basal dose of N. The experiment was carried out till harvest of paddy crop (120 days) in completely randomized design. The soil without biochar treatment served as control for each experiment. Biometric observations such as plant height, shoot dry weight, root dry weight and root length were recorded after the harvest. Soil samples were analysed after the harvest of crop for the pH, EC, exchangeable acidity, exchangeable Ca, available Si along with soluble and exchangeable Al.

### Chemical analysis

The soil pH and EC were measured in a soil–water suspension with 1:2.5 ratio^48^. Soil was treated with 1 N KCl, shaken for minutes, filtered, and titrated against 0.1 N NaOH using phenolphthalein as indicator for measuring exchangeable acidity of soil. Extraction of soil with 1 N (pH 7) ammonium acetate followed by complexometric titration was followed for exchangeable Ca^52^. Soil was extracted with 0.5 M acetic acid, the plant available Si in the extract was determined using UV- visible spectrophotometer at 630 nm^53^ (Shimadzu Pharma UV^−1^,700 UV visible spectrophotometer).

Soil was treated with 0.02 M CaCl_2_ in 1:5 ratio for soluble Al estimation. After continuous end to end shaking in a mechanical shaker for 1 h, the solution was centrifuged at 2,500 rpm for 10 min and then filtered. Sequentially 1 N KCl was added to soil in 1:5 ratio for exchangeable Al estimation and after shaking for 30 min, it was centrifuged at 2,500 rpm for 10 min and filtered. Aluminium in the filtrate was determined by measuring the absorbance at 395 nm using UV visible spectrophotometer^54^.

### Statistical analysis

The experiment was performed with three replicates. All the data were analysed using one-way ANOVA with SPSS software (IBM Corp. Released 2013. IBM SPSS Statistics for Windows, Version 21.0. Armonk, NY: IBM Corp). Data obtained from the studies were subjected to Tukey’s HSD post-hoc test for comparing mean difference between treatments. All the statistical analyses were done at *p* ≤ 0.05 level of significance.
